# GlyNAC (Glycine and *N*-Acetylcysteine) Supplementation Improves Impaired Mitochondrial Fuel Oxidation and Lowers Insulin Resistance in Patients with Type 2 Diabetes: Results of a Pilot Study

**DOI:** 10.3390/antiox11010154

**Published:** 2022-01-13

**Authors:** Rajagopal V. Sekhar

**Affiliations:** Translational Metabolism Unit, Section of Endocrinology, Diabetes and Metabolism, Baylor College of Medicine, Houston, TX 77030, USA; rsekhar@bcm.edu

**Keywords:** GlyNAC, glycine, *N*-acetylcysteine, mitochondria, insulin resistance, type 2 diabetes

## Abstract

Patients with type 2 diabetes (T2D) are known to have mitochondrial dysfunction and increased insulin resistance (IR), but the underlying mechanisms are not well understood. We reported previously that (a) adequacy of the antioxidant glutathione (GSH) is necessary for optimal mitochondrial fatty-acid oxidation (MFO); (b) supplementing the GSH precursors glycine and *N*-acetylcysteine (GlyNAC) in mice corrected GSH deficiency, reversed impaired MFO, and lowered oxidative stress (OxS) and IR; and (c) supplementing GlyNAC in patients with T2D improved GSH synthesis and concentrations, and lowered OxS. However, the effect of GlyNAC on MFO, MGO (mitochondrial glucose oxidation), IR and plasma FFA (free-fatty acid) concentrations in humans with T2D remains unknown. This manuscript reports the effect of supplementing GlyNAC for 14-days on MFO, MGO, IR and FFA in 10 adults with T2D and 10 unsupplemented non-diabetic controls. Fasted T2D participants had 36% lower MFO (*p* < 0.001), 106% higher MGO (*p* < 0.01), 425% higher IR (*p* < 0.001) and 76% higher plasma FFA (*p* < 0.05). GlyNAC supplementation significantly improved fasted MFO by 30% (*p* < 0.001), lowered MGO by 47% (*p* < 0.01), decreased IR by 22% (*p* < 0.01) and lowered FFA by 25% (*p* < 0.01). These results provide proof-of-concept that GlyNAC supplementation could improve mitochondrial dysfunction and IR in patients with T2D, and warrant additional research.

## 1. Introduction

Type 2 diabetes (T2D) is associated with mitochondrial dysfunction [1,2] which involves the impaired oxidation of fatty-acids (FA) [3,4,5,6,7,8,9,10,11,12,13]. Mitochondria are the source of energy generation and reactive oxygen species in cells, and mitochondrial dysfunction has been linked to diabetic complications [14,15] involving the heart [15,16,17,18,19,20], skeletal muscle [3,4,5,6,7,20], kidneys [21,22] and liver [23,24], and is also associated with inflammation [25], aging [26], cognitive disorders [27,28] and insulin resistance (IR) [29,30]. During the process of energy generation, mitochondria generate toxic reactive oxygen species (ROS) which induce a harmful state known as oxidative stress (OxS). To defend against OxS, mitochondria critically depend on antioxidants for protection, and glutathione (GSH, γ-glutamyl-cysteinyl-glycine) is the most abundant endogenous intracellular antioxidant [31,32]. Acute depletion of intracellular GSH concentrations result in mitochondrial injury or irreversible cell damage [16,33], suggesting that GSH is important for mitochondrial function and survival. GSH is a tripeptide synthesized from glutamic acid, cysteine and glycine in two steps catalyzed by the enzymes glutamate cysteine ligase (GCL, also known as γ-glutamylcysteine synthetase) and γ-l-glutamyl-l-cysteine:glycine ligase (also known as glutathione synthetase), and is important in health and disease [31,32]. Patients with T2D are reported to have GSH deficiency [34,35,36,37,38,39]. In a pilot study conducted earlier, we investigated the mechanisms contributing to GSH deficiency in T2D and reported that diminished synthesis contributes to GSH deficiency, and that this occurs due to decreased availability of the GSH precursor amino acids glycine and cysteine, and not glutamic acid [39]. We also reported that supplementing these patients with T2D with glycine and cysteine (provided as *N*-acetylcysteine, NAC) for a short duration of 2 weeks corrected the impaired GSH synthesis, improved intracellular GSH concentrations, and lowered OxS, without a decrease in fasting blood glucose concentrations [39]. This combination of glycine and NAC is abbreviated as GlyNAC. In rodent studies, we discovered that depleting GSH in young healthy mice results in impaired mitochondrial fatty-acid oxidation (MFO) [40], and that supplementing GlyNAC in old mice corrected GSH deficiency, reversed impaired MFO and lowered IR [40]. The results of these rodent studies suggest that GSH adequacy is critically important for optimal and efficient mitochondrial function, and that GlyNAC supplementation could be important for improving mitochondrial dysfunction and lowering IR. We tested this in human clinical trials involving older humans and in HIV-infected patients, and found that GlyNAC supplementation for a short duration of 2 weeks was sufficient to improve mitochondrial dysfunction and lower IR in both trials [41,42], but also that GlyNAC supplementation over longer durations ranging from 12–24 weeks reversed, corrected and normalized mitochondrial dysfunction compared to controls, also further improved IR [43,44]. However, stopping GlyNAC supplementation in both trials resulted in a recurrence of mitochondrial dysfunction and worsening of IR [43,44]. These observations indicate causality where defects improve with GlyNAC supplementation, and recur after stopping GlyNAC. Next, we tested and found that GlyNAC supplementation in old mice reversed impaired MFO in the heart and improved cardiac function [45]. However, whether GlyNAC supplementation can improve mitochondrial dysfunction or IR in patients with T2D remains unknown.

Reversing mitochondrial impairment is a key focus in T2D [46]. The effect of GlyNAC on mitochondrial function in patients with T2D has not been previously reported in the medical literature. This manuscript reports unpublished data on mitochondrial fuel oxidation, insulin resistance and free-fatty acid (FFA) concentrations from a previous study investigating the effect of supplementing GlyNAC in patients with T2D [39].

## 2. Materials and Methods

### 2.1. Study Approval

The study was conducted in accordance with the Declaration of Helsinki, and the protocol was approved by the Institutional Review Boards (IRB) at Baylor College of Medicine. The clinicaltrials.gov results database was made available to the public in September 2008; this study began in 2004 and was completed before 2008.

### 2.2. Study Participants

This manuscript contains unpublished results (from our earlier published study [39]) on mitochondrial, IR and FFA data from a subset of 10 adults with poorly controlled T2D (HbA1c 8–10%) and 10 non-diabetic controls. Participants did not have any thyroid disorder, hypercortisolemia, liver or renal impairment, malignancy, active infections, steroid therapy, and did not have any hospitalizations in the 6 months prior to study participation. No participants consumed any dietary supplements or alcoholic beverages. All diabetic patients were under the care of their primary physicians. To prevent acute swings of blood glucose and to achieve comparable glycemic levels before and after supplementation with GlyNAC, only newly diagnosed diabetic patients who were not receiving insulin therapy were recruited, and all diabetic participants were being treated with lifestyle modification ± oral antidiabetic agents only.

### 2.3. Study Protocol

Patients with T2D were studied before and 2 weeks after receiving oral supplementation for 14 days (2 weeks) with GlyNAC (combination of glycine and *N*-acetylcysteine (NAC, as a cysteine donor)) [39]. Participants were studied after an overnight fast with collection of blood and urine, and underwent indirect calorimetry after a fast duration of 18 h. The diabetic participants underwent the study protocol before and after 14 days of supplementation. Glycine and NAC capsules were prepared by a pharmacist and dosed to provide 100 mg/kg/day of glycine, and 100 mg/kg/day of NAC. Compliance was assessed by phone calls and counting of capsules at the end of the 2-week period. Control subjects did not receive GlyNAC supplementation.

### 2.4. Outcome Measures

#### 2.4.1. Mitochondrial Function 

Mitochondrial function was assessed using indirect calorimetry (Deltatrac, Sensormedics, Fullerton, CA, USA), with measurement of oxygen consumption and carbon dioxide production. Respiratory quotient (RQ) is the ratio of directly measured carbon dioxide produced to oxygen consumed, and denotes the type of fuel substrate being oxidized. Mitochondrial fatty-acid oxidation (MFO) and mitochondrial glucose oxidation (MGO) were calculated using calorimetric data [47].

#### 2.4.2. Glycemic and Lipid Analyses

Plasma insulin concentrations were measured using a commercially available, highly specific radioimmunoassay kit for human insulin (Linco Research, St. Charles, MO, USA). Insulin resistance was calculated (as HOMA-IR) as reported by us in prior studies [42,43,44]. Plasma free-fatty acid concentrations were measured using a spectrophotometric assay (Wako Chemicals, Neusse, Germany). 

### 2.5. Statistical Analyses

Data are expressed as means ± SE. A repeated measures analysis of variance (ANOVA) with the Bonferroni multiple comparisons test was used for the statistical analyses to compute differences in means between the diabetic group pre-supplementation and the control group, and in the diabetic patients studied pre- and post-supplementation. Results were considered to be statistically significant at *p* < 0.05. 

## 3. Results

### 3.1. Age and Body Mass Index (BMI)

There were no significant differences between nondiabetic controls and diabetic patients for age (*p* = 0.99). Comparisons of BMI between the 2 groups did not show any differences between controls or T2D patients pre-supplementation (*p* = 0.3), or in T2D patients pre- and post- GlyNAC supplementation for 2 weeks (*p* > 0.99) (Table 1).

### 3.2. Mitochondrial Function

**Figure 1:**
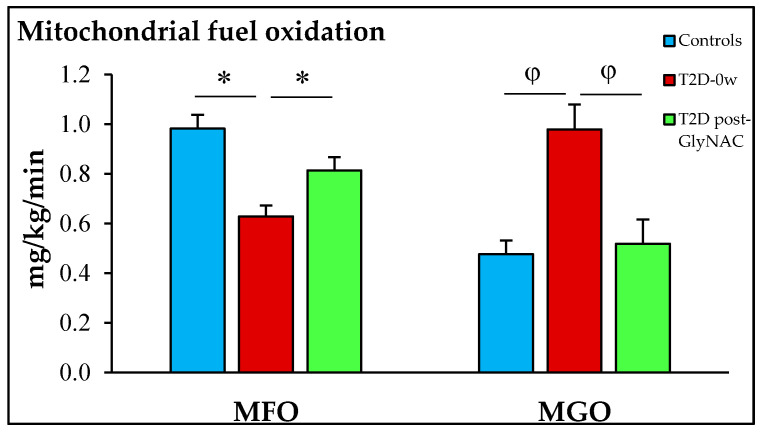
GlyNAC supplementation improves impaired mitochondrial fuel oxidation in patients with T2D. T2D = type 2 diabetes; MFO = mitochondrial fatty-acid oxidation; MGO = mitochondrial glucose oxidation; T2D-0w = T2D patients before GlyNAC supplementation; T2D post-GlyNAC = T2D patients 2-weeks after GlyNAC supplementation. ∗ = *p* < 0.001; φ = *p* < 0.01.

Compared to fasted non-diabetic controls, fasted diabetic participants had 36% lower MFO (*p* = 0.0006) and 106% higher MGO (*p* = 0.008). GlyNAC supplementation was associated with a 30% increase in MFO (*p* = 0.0009), and a 47% decrease in MGO (*p* = 0.001) (Figure 1) indicating an improvement in impaired fasted mitochondrial fuel oxidation. (Table 1). In this study there were no gender differences, but future studies with larger sample sizes are needed to more accurately evaluate gender differences.

### 3.3. Glycemia, Insulin Resistance and Plasma Free Fatty-Acid Concentrations

**Figure 2:**
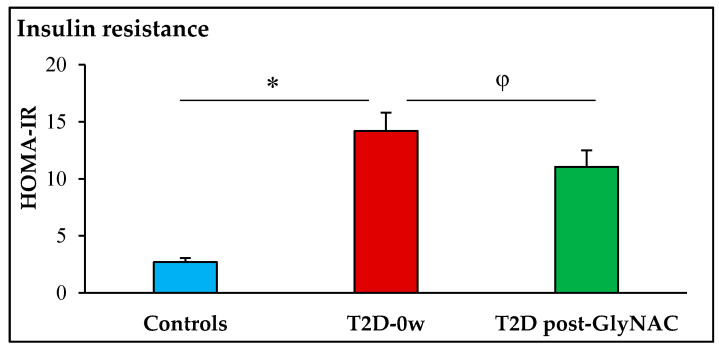
GlyNAC supplementation lowers insulin resistance in participants with Type 2 diabetes. HOMA-IR = homeostatic model assessment for insulin resistance; T2D-0w = participants with Type 2 diabetes before GlyNAC supplementation; T2D post-GlyNAC: participants with T2D 2 weeks after GlyNAC supplementation. ∗ = *p* < 0.001; φ = *p* < 0.01.

**Figure 3:**
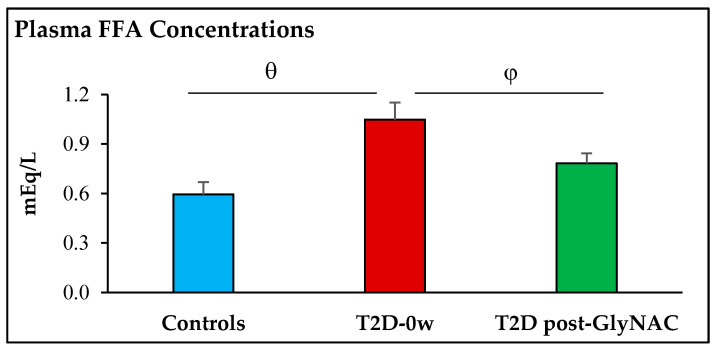
GlyNAC supplementation lowers plasma free-fatty acid concentrations in Type 2 diabetes. FFA = free fatty acids; T2D-0w = participants with Type 2 diabetes before GlyNAC supplementation; T2D post-GlyNAC: participants with T2D participants 2 weeks after GlyNAC supplementation. φ = *p* < 0.01; θ = *p* < 0.05.

Fasting participants with T2D had 103%, 160% and 76% higher concentrations of plasma glucose (*p* < 0.0001), insulin (*p* = 0.0002) and FFA (*p* = 0.015) respectively, and 425% higher insulin resistance (*p* = 0.0002) compared to non-diabetic controls. GlyNAC supplementation significantly lowered fasting plasma concentrations of insulin by 19% (*p* = 0.0006), FFA by 25% (*p* = 0.004), and decreased IR by 22% (*p* = 0.006), but there were no improvements in fasting plasma glucose concentrations (*p* = 0.7) (Table 1, Figure 2 and Figure 3).

## 4. Discussion

Patients with T2D are known to have impaired mitochondrial fuel oxidation [1,2,3,4,5,6,7,8,9] and insulin resistance [29,30], and these defects were present in the diabetic participants in this study. The novel discovery in this study is that GlyNAC supplementation improves defects in fasting mitochondrial fatty-acid and glucose oxidation, and lowers insulin resistance and plasma free fatty-acid concentrations within a short duration of 2 weeks.

### 4.1. GlyNAC Supplementation Improves Mitochondrial Impairment in T2D

Previous studies have shown that diabetes is associated with impaired oxidation of fatty-acids [3,4,5,6,7,8,9,10,11,12,13,14,15] but reversing this defect in humans with T2D has been difficult to achieve. In an earlier study we investigated and found that depleting GSH concentrations in young healthy mice using an inhibitor of GSH synthesis resulted in impaired mitochondrial fatty-acid oxidation [40], which suggests that adequacy of GSH is essential for mitochondrial fatty-acid oxidation. Next, we investigated old mice which had GSH deficiency in tissues (liver and skeletal muscle) and also had impaired mitochondrial fatty-acid oxidation. Supplementing GlyNAC in these old mice (but not isonitrogenous-isocaloric placebo) corrected tissue GSH deficiency, and also corrected mitochondrial fatty-acid oxidation which was measured using calorimetry, tracers and molecular expression of regulators of substrate oxidation [40]. Of particular relevance is the measurement of respiratory quotient (RQ) which is sometimes also referred to as the respiratory exchange ratio (RER). The process of complete substrate oxidation requires the consumption of oxygen and production of carbon dioxide, and RQ is the ratio of carbon-dioxide produced and oxygen consumed during respiration. For example, complete oxidation of a fuel substrate such as glucose (C_6_H_12_O_6_) is depicted by the equation C_6_H_12_O_6_ + 6O_2_ → 6CO_2_ + 6H_2_O, and shows that oxidation of one mole of glucose requires the consumption of 6 moles of oxygen and production of 6 moles of carbon dioxide. In this reaction, the ratio of CO_2_ produced to O_2_ consumed (i.e., the RQ) is 6/6 = 1. Each fuel substrate has its own signature RQ, and similarly to how the RQ for glucose oxidation is 1.0, the RQ of fatty-acid oxidation is around 0.7. The RQ depicts the relative composition of glucose or fatty acids in the fuel mix being oxidized, and is a direct measure based on gas exchange. In our published studies in old mice, old humans and HIV-patients we found evidence of impaired mitochondrial fatty-acid oxidation and that this was improved/corrected after supplementation with GlyNAC [43,44]. Stopping GlyNAC supplementation in our human studies resulted in a loss of benefits, with a recurrence of mitochondrial defects [43,44]. These previous reports that GSH adequacy is critically important for optimal mitochondrial fatty-acid oxidation [40], that GlyNAC supplementation corrects GSH deficiency and improves mitochondrial fatty-acid oxidation [40,42,43,44], and that these defects recur after GlyNAC withdrawal [43,44], suggest causality. In our study in HIV-infected patients, we found and reported that GlyNAC supplementation also improved mitochondrial defects in skeletal muscle, including improvements in impaired expression of PGC1α (mitochondrial biogenesis) and PINK1 (mitophagy) [43]. It is against this background that the results reported in this manuscript are significant, because GlyNAC supplementation in participants with T2D improved RQ, MFO and MGO in a relatively short duration of 14 days. These abnormalities in fuel oxidation and gas exchange have been identified as predictors of weight gain [48], and this could be of particular importance in T2D which is strongly associated with weight gain, termed by some as ‘diabesity’. Because GlyNAC supplementation improves fuel metabolism, it will be important to study its effects on body composition including liver fat content in future studies. Overall, these results suggest that GlyNAC supplementation could improve mitochondrial health in patients with T2D, and support the need for future clinical trials to investigate the impact of GlyNAC supplementation on improving the health of diabetic patients. 

### 4.2. Effect of GlyNAC Supplementation on Fasting Plasma Insulin and Free-Fatty Acid Concentrations, and Insulin Resistance in Diabetes

Fasting hyperinsulinemia and elevated IR are pathognomonic defects in T2D. IR is associated with mitochondrial dysfunction [29,30]. Compared to nondiabetic controls, diabetic participants in this study had severely elevated fasting insulin concentrations (160% higher) and IR (425% higher), and they decreased by 19% and 22% respectively after 2 weeks of supplementation with GlyNAC. However, this decrease in IR was modest (but significant) and did not come down to levels found in nondiabetic controls; the most likely reason for this was the short duration of supplementation. Nonetheless, the fact that just 2 weeks of GlyNAC supplementation was sufficient to significantly lower insulin resistance by 22% is exciting, and future studies are needed to investigate the effect of longer durations of GlyNAC supplementation on whole-body, skeletal muscle, and hepatic insulin resistance in patients with T2D. These data are congruent with our earlier publication where we found that GlyNAC supplementation in aged mice improved abnormalities in glucose and insulin tolerance testing to match levels in young mice [40].

Why does insulin resistance improve with GlyNAC? There could be multiple reasons to explain this. Insulin resistance is associated with mitochondrial dysfunction, elevated circulating free-fatty acid levels, and increased OxS. GlyNAC supplementation improved all of these defects in this study, and this could have contributed to the improved insulin resistance. Elevated plasma fatty-acid concentrations have also been linked to insulin resistance [49,50,51]. A randomized clinical trial reported that lowering free-fatty acids did not improve oxidative capacity in T2D [52]. However, in this study GlyNAC supplementation lowered plasma FFA levels and improved mitochondrial fuel oxidation, suggesting that both defects improved simultaneously in response to GlyNAC, but whether these defects are mechanistically interlinked is unclear and needs further study. 

Fasting hyperinsulinemia, in particular, is a key defect in T2D and occurs in response to progressive increases in IR. For this to occur, there is β-cell hyperactivity to secrete higher amounts of insulin to overcome the IR; over time this results in β-cell burnout and the need for insulin replacement. Therefore, it is clear that IR is directly linked to β-cell stress (i.e., hyperactivity) and contributes to β-cell failure over time. If this negative spiral could be broken, there could be less stress on β-cells with a possibility of longer β-cell survival. We tested this in mice, where we found that aged mice had higher insulin and glucose responses to a glucose challenge together with blunted glucose lowering in response to an insulin challenge, indicating that the aged mice had increased β-cell activity and IR (impaired insulin-stimulated glucose disposal) [40]. When these aged mice received GlyNAC, their insulin-stimulated glucose disposal improved dramatically to match values in young, healthy mice and their glucose response to a glucose tolerance test also showed a striking improvement to match healthy, young control mice. However, the interesting finding was a significant decline in the hyperinsulinemic response to a glucose tolerance test, suggesting less stress on β-cells. Seen together, these rodent data indicate that GlyNAC supplementation lowered IR and improved insulin sensitivity, and therefore less insulin was needed for glucose disposal, relieving stress on β-cells. When the results of this pilot study are viewed against the background of these rodent data, a similar pattern can be recognized. Patients with T2D had elevated fasting glucose and insulin concentrations, indicating higher IR and increased β-cell activity. GlyNAC supplementation significantly lowered both fasting insulin concentrations and IR, without lowering fasting hyperglycemia or HbA1c. If the fall in insulin concentration was due to β-cell failure, and not in response to a drop in IR, there would have been a rise in hyperglycemia, but this did not occur. The most parsimonious explanation is that with lowering of insulin resistance (and thereby improving insulin sensitivity), glucose disposal could be achieved with less insulin, and this reduced β-cell overactivity. These data suggest the real and exciting possibility that GlyNAC supplementation could promote β-cell health by preventing β-cell stress and overactivity, and more studies focused on the β-cell effects of GlyNAC supplementation are needed. Mitochondrial dysfunction has been reported to adversely affect the β-cell function [53,54,55,56,57], and it is possible that an additional mechanism by which GlyNAC could have improved β-cell health and function is by improving mitochondrial function in β-cells, but this remains to be proven. Therefore, GlyNAC supplementation in T2D could have implications for glucose metabolism beyond lowering insulin resistance, and this should be investigated in future studies. 

### 4.3. Salient Aspects of GlyNAC-Mediated Improvements in Mitochondrial Fuel Oxidation and Insulin Resistance in T2D

There are several important aspects of the GlyNAC-mediated improvements of these two key outcomes in diabetes: *(a) Speed of onset:* GlyNAC supplementation acts quickly with significant improvements occurring after just 2 weeks. This speed of improvement is consistent with the observed effects of GlyNAC in our other human studies in older humans and HIV patients where 2 weeks of GlyNAC supplementation improved MFO, MGO and IR; *(b) Improvements occur despite the presence of hyperglycemia in patients with T2D:* 2 weeks of time is too short to observe meaningful changes in glycosylated hemoglobin concentrations, which remained unchanged in this study. There were no changes in fasting glucose concentrations; the overall picture suggests that hyperglycemia remained relatively unchanged during this time. Because diabetic benefits typically depend on lowering hyperglycemia and these improvements occurred without a change in hyperglycemia, these findings suggest that alternate mechanisms (other than glycemic control) could be involved in improving mitochondrial function and IR in this study; *(c) Magnitude of response*: GlyNAC supplementation did not ‘normalize’ mitochondrial defects or insulin resistance to levels found in controls, and the most likely reasons for this are the short 2 week duration of supplementation and the presence of uncontrolled hyperglycemia. In future studies, it will be interesting to evaluate whether longer durations of GlyNAC supplementation could result in greater improvements in MFO, MGO and IR, and if benefits decline after GlyNAC withdrawal.

### 4.4. Why GlyNAC Works—The ‘Power of Three’

GlyNAC supplementation provides glycine, cysteine (from NAC) and GSH (from glycine and cysteine) [39,41,42]. Glycine is of vital importance to cellular health as a 1-carbon metabolite and methyl (CH_3_) group donor [58,59]. Glycine and methyl groups are required by multiple cellular pathways for the synthesis of important metabolites and metabolic intermediates, such as purines for DNA synthesis [58,59,60,61]. Glycine acts as a neurotransmitter in the brain [62,63,64,65,66] and is an important component of cartilage [67]. Cysteine contains a sulfhydryl (SH) group donor and is an important thiol in antioxidant systems [68] and for multiple cellular processes, especially in mitochondria [69,70,71]. For example, coenzyme A (CoA-SH) is an important intermediate in the mitochondrial β-oxidation of fatty-acids and for pyruvate metabolism in the Krebs’ cycle, and requires cysteine for its synthesis [72]. Cysteine is also important in maintaining protein structure, iron metabolism and other reactions in the body [71,73,74]. A key function of both glycine and cysteine is to serve as precursors for the synthesis of glutathione, the most abundant endogenous, intracellular tripeptide antioxidant [31,32]. GSH is commonly referred to as the ‘master antioxidant’, both for its abundance and for the multitude of biological functions that it supports [31,32,75,76]. GSH combats OxS, provides cellular protection, is required for efficient mitochondrial function, participates in detoxification of harmful metabolites, supports glutathionylation function, and is important for multiple and varied cellular processes [31,32,76,77]. We have termed the beneficial effects of the combination of glycine, cysteine and glutathione as the ‘power of three’ [43,44,78] because they act rapidly (after 2 weeks of GlyNAC supplementation in this study) to provide a powerful biological effect toward cellular protection, correcting cellular defects and improving cell, organ and organism health. 

### 4.5. Study Limitations

The key limitations of this study are a small sample size, lack of a placebo group, and the need for data at the molecular level. Nonetheless, these data from our exploratory pilot study suggests that GlyNAC supplementation could be of key importance in improving mitochondrial health, dyslipidemia and insulin resistance in humans with T2D. However, it is important to recognize that these exciting results require confirmation in a randomized clinical trial.

## 5. Conclusions

The results of this exploratory pilot study suggest that supplementing GlyNAC in patients with T2D could improve defects in mitochondrial function, lower insulin resistance and circulating plasma fatty-acid concentrations. These results could have important implications for improving health in diabetes, and support the need for a randomized clinical trial to confirm these findings and to understand the impact of GlyNAC supplementation on defects linked to mitochondrial dysfunction in diabetes.

## Figures and Tables

**Table 1:** **Fasted fuel oxidation, glycemic indices and FFA concentrations.** Values are means ± SE; Means are significantly different at *p* < 0.05. T2D = type 2 diabetes; 0-weeks = baseline study; 2-weeks = study conducted after 2-weeks of GlyNAC supplementation; FFA = free-fatty-acids, HOMA-IR = insulin resistance.

**Parameter**	**Non-Diabetic Controls: 0-weeks**	**Diabetic Patients: 0-weeks** *Controls* vs. *T2D-0-weeks*	**Diabetic Patients: 2-weeks** *T2D-0-weeks* vs. *T2D-2-weeks*
Age (years)	50.8 ± 5.0	50.9 ± 4.4 *p = 0.99*	-
Glycosylated hemoglobin (HbA1c)	5.5 ± 0.1	9.2 ± 0.2 *p < 0.0001*	9.1 ± 0.3 *p > 0.99*
Body mass index (BMI)	27.7 ± 0.4	29.9 ± 0.9 *p = 0.3*	29.7 ± 1.0 *p > 0.99*
Fasting respiratory quotient (RQ)	0.76 ± 0.00	0.81 ± 0.01 *p = 0.02*	0.77 ± 0.01 *p = 0.001*
Fasting FA oxidation (mg/kg/min)	0.98 ± 0.06	0.63 ± 0.04 *p = 0.0006*	0.81 ± 0.05 *p = 0.0009*
Fasting glucose oxidation (mg/kg/min)	0.48 ± 0.06	0.98 ± 0.10 *p = 0.008*	0.68 ± 0.08 *p = 0.001*
Fasting plasma glucose (mmol/L)	5.5 ± 0.2	11.2 ± 0.5 *p < 0.0001*	10.8 ± 0.5 *p = 0.7*
Fasting insulin concentrations (pmol/L)	11.0 ± 1.4	28.5 ± 0.9 *p = 0.0002*	23.1 ± 2.6 *p = 0.0006*
Insulin resistance (HOMA-IR)	2.7 ± 0.3	14.2 ± 1.6 *p = 0.0002*	11.1 ± 1.4 *p = 0.006*
Fasted plasma FFA (mEq/L)	0.59 ± 0.07	1.02 ± 0.11 *p = 0.015*	0.76 ± 0.07 *p = 0.004*

## Data Availability

All of the data is contained within the article.
